# Mitochondrial disorders as a mechanism for the development of obese Sarcopenia

**DOI:** 10.1186/s13098-023-01192-w

**Published:** 2023-11-06

**Authors:** Tingfeng Liao, Lijiao Xiong, Xiaohao Wang, Shu Yang, Zhen Liang

**Affiliations:** 1 School of Medicine, The First Affiliated Hospital of Southern University of Science and Technology, Shenzhen People’s Hospital (The Second Clinical Medical College, Jinan University), Shenzhen, China; 2 Department of Geriatrics, Shenzhen People’s Hospital, (The Second Clinical Medical College, Jinan University, The First Affiliated Hospital, Southern University of Science and Technology, Shenzhen, China; 3 Shenzhen Clinical Research Centre for Geriatrics, Shenzhen People’s Hospital, Shenzhen, China

## Abstract

Obese sarcopenia is a severe and prevalent disease in an aging society. Compared to sarcopenia alone, the development and advanced stage of obesity sarcopenia is faster and more severe. Diagnosis of the cause of adipocyte accumulation is also more complicated; however, no effective pharmacological treatment is available. Chronic inflammation is one of the causes of sarcopenia, and obese patients, who are more likely to develop chronic inflammation, may simultaneously suffer from obesity and sarcopenia. Mitochondrial metabolic disorders have been more easily observed in the tissue cells of patients with obesity and sarcopenia. Mitochondrial metabolic disorders include abnormal mtDNA release, mitochondrial autophagy, and dynamic mitochondrial disorders. Therefore, this review will reveal the mechanism of development of obesity myasthenia gravis from the perspective of mitochondria and discuss the currently existing small-molecule drugs.

## Obese sarcopenia is one of the most severe types of Sarcopenia

Sarcopenia, an age-associated reduction in skeletal muscle mass and function, is a recognized risk factor for adverse health conditions, including disability, frailty, institutionalization, and mortality [1]. The development of sarcopenia may be accompanied by obesity, which is more prevalent in older adults worldwide [2]. The global prevalence of obesity is high [3]. Obese patients with sarcopenia have an increased risk of metabolic syndrome and movement disorders, decreasing their ability to care for themselves [4]. The skeletal muscle is one of the essential organs in the body. Many negative factors, including aging, inflammation, and high blood glucose levels, seriously injure obese patients with sarcopenia [2, 5, 6]. Sarcopenia can occur with besity in any age group, which is attributed to the harmful effects of adipose tissue-related metabolic disorders such as oxidative stress, inflammation, and insulin resistance; therefore, obesity can independently cause decreased skeletal muscle mass and function [7–9]. The risk of body organ dysfunction caused by this complex disease is 2–3 times higher than that caused by sarcopenia or obesity alone [2]. Obese sarcopenia, a combination of sarcopenia and obesity, in addition to the characteristics of sarcopenia, also presents an increase in fat mass, which makes diagnosis more difficult [10]. The definition of obese sarcopenia is not yet universally established. However, various diagnostic criteria have been established for sarcopenia and obesity, which reveals that although popular, it is not receiving enough attention.

### The mechanisms of obesity and sarcopenia

The mechanism of obesity and sarcopenia are interlinked and inseparable. Skeletal muscle myocytes express and secrete cytokines such as IL-8, IL-6, irisin and myostatin [11]. Myokines may affect adipocytes cells via endocrine effects [12–15]. In a healthy body environment, adipose cells and muscle cells will secrete appropriate secretion factors to promote each other, to ensure the normal function and number of satellite cells, to ensure the regeneration of muscle cells. White adipose tissue would express leptin, IL-6, TNF-α and resistin systemically during an exacerbation environment, which are involved in various signaling cascades and regulating skeletal muscle protein balance [16–18]. Fatty acids from dysfunctional adipocytes can also affect skeletal muscle stability. Clinical studies have shown that obese people have more fat infiltration in other organs of the body, especially in the skeletal muscle [19]. Notch signaling and Wnt signaling responses in skeletal muscle cells regulate satellite cell proliferation and muscle regeneration through the influence of fat [20–23]. It is interesting that fatty infiltration and muscle fibrosis can impair muscle quality without atrophy; in these situations, muscle mass may not actually change. Ectopic lipid deposition in hyperlipidemia is often associated with dysregulation of mitochondrial lipid oxidation in adipose patients because of increased ROS and inflammatory factors, two mechanisms that combine to make sarcopenia more likely [16, 24]. Physical activity can slow down inflammation and lipid deposition in the body, and through the notch signaling pathway, it can help establish balance between fat and skeletal muscle at the same time according to clinical studies [25–27]. Similarly, cold stimulation can also accelerate metabolism and build muscle through the activation of brown fat, and maintain mitochondrial integrity and functional recovery while being detected [28–30]. Many signal pathways under cold stimulation and exercise would benefit skeletal muscle and systemic metabolism, such as usp21 [31, 32]. In short, both adipose tissue and skeletal muscle tissue are secretory organs that can communicate and can respond to changes in the environment by receiving signals and simultaneously producing changes.

### Mitochondria are the key organelles of cell metabolism

Aerobic respiration involves three processes in living cells, and mitochondria are critical organelles that produce a large quantity of ATP that is transported to reactions requiring energy and play a key role in maintaining metabolic activity in the body. Changes in the extracellular environment induce dynamic changes in mitochondria, increasing or decreasing enzymatic activity [33]. Dysfunctional mitochondria are associated with many metabolic disorders, and mitochondria are metabolic organelles that act centrally in lipid and protein synthesis and are related to obese sarcopenia [34, 35]. Mitochondrial dysfunction leads to increased production of reactive oxygen species (ROS), leading to chronic low-grade inflammation and impaired metabolism of muscle proteins and affecting the energy supply [36].

### Mitochondria can interfere with obese Sarcopenia

Mitochondrial dysfunction is more detrimental in obese sarcopenia than in mono-sarcopenia or mono-obesity because of the more significant negative impact of crosstalk between the skeletal muscle and adipose tissue. Dysregulated protein metabolism impedes average mitochondrial turnover, rapidly accumulating dysfunctional mitochondria and increased damage, further exacerbating organelle and tissue dysfunction in a vicious cycle [34]. In a zebrafish model of obese sarcopenia, muscle atrophy was associated with obese sarcopenia-induced mitochondrial dysfunction [37]. During obesity, the accumulation of bioactive lipids in the skeletal muscle intermediates propagates mitochondrial dysfunction. Altered mitochondrial bioenergetics and turnover targeted by dietary regimens and exercise hinder the age-related accumulation of ROS and increase in systemic chronic inflammatory factors, as medical recommendations to target sarcopenia and obesity, thereby maintaining muscle protein metabolic homeostasis and reflecting on mitochondrial health [34]. The maintained protein turnover rapidly removes damaged abnormal proteins, such as dysfunctional mitochondria and contractile proteins, while synthesizing new functional proteins normally, resulting in the preservation of mitochondrial mass, muscle mass, and strength. In summary, mitochondrial quality is associated with muscle quality and can be used to predict disease occurrence [38].

This review will conclude the interaction between mitochondria and obese sarcopenia and show the risk factors of abnormal mitochondria.

## Mitochondrial DNA

### Mitochondrial DNA is easily damaged in the case of Disease

Mitochondrial DNA (MtDNA) is usually a tiny double-stranded circular molecule located in the mitochondrial matrix to maintain the normal metabolism of mitochondria, which can encode 13 oxidative phosphorylated mRNA, tRNA, and ribosomal RNA, as well as enzymes required for transcription and translation in the mitochondrial matrix [39]. Indeed, mtDNA is not a “naked” pure nucleic acid but is packaged into protein–DNA complexes called nucleotides and maintains stability even under normal conditions. MtDNA-binding protein transcription factor A (TFAM), the transcriptional activator initially identified as the MtDNA promoter in humans and mice, is the main component that initiates and drives MtDNA packaging and the overall ribosome structure and is the main component that maintains the stability of mtDNA function and structure. The concentration of TFAM and its binding density to mtDNA are likely to be regulated to achieve different packaging patterns and precise tuning of mtDNA transcription. Generally, TFAM packaging may partially protect mtDNA from oxidative damage, a mode of protection enhanced by a robust mitochondrial base excision repair pathway in response to oxidative and other non-bulky base damage [40]. One study found that reducing TFAM in mouse adipose tissue reduces mtDNA copy number and leads to metabolic diseases like diabetes. Thus, TFAM knockout mice exhibited higher energy expenditure and were protected from age- and diet-induced obesity, insulin resistance, and hepatic steatosis [41]. Therefore, mammalian mtDNA does not lack a protective or repair mechanism. The specific stable amount of cell mtDNA damage is the balance between the amount of damage sustained in the mitochondrial matrix oxidation environment caused by environmental stress and the efficiency of mtDNA repair. According to existing research, sports and healthy diet can accelerate mtDNA repair efficiency [40, 41].

### The occurrence of Sarcopenia is accompanied by mitochondrial disorders

The skeletal muscle has high demand and a high density of mitochondria. The occurrence of sarcopenia is usually accompanied by the damage of mtDNA of skeletal muscle [42, 43]. Mitochondria are strong promoters of inflammation caused by sterile injury and regulate antibacterial inflammation [44]. Mutations and an abnormal release of mtDNA may lead to severe diseases. Mitochondria are the source of intracellular oxidants and the main target of oxidative stress in cells. According to studies on mitochondria, the absence of histones and introns makes it challenging to repair mtDNA quickly in the presence of additional oxidative stress damage. According to a recent study, mtDNA fragments in the cell membrane are transferred to the nucleus under abnormal conditions [45]. MtDNA can be inserted into nuclear DNA, leading to the instability of the entire genome, which in turn has a certain probability of affecting the normal functional state of the entire cell or tissue. Nuclear localization of mtDNA fragments may also affect nearby centromeres and induce chromosomes, which may affect the organism’s normal development [46]. Therefore, an abnormal rupture of a single mitochondrion will cause damage to the whole cell or even tissue, leading to sarcopenia [47, 48]. Some researches showed that Resveratrol, a drug that prevents high-fat diet-induced muscle atrophy in aged rats by reversing mitochondrial dysfunction and reducing oxidative stress, could treat the disease by restoring mtDNA copy number and reversing the expression of mitochondria-associated proteins in vitro via the PKA/LKB1/AMPK signaling pathway [49–52].

### Obesity could be the cause of abnormal mtDNA

The factors that induce chronic low-grade inflammation are abnormalities, such as obesity, which increase the risk of cancer, diabetes, and sarcopenia [52, 53]. The oxidative stress was higher in the obesity model than in the normal model, which revealed that obesity is associated with ROS [54]. More and more reliable data provide high evidence that oxidative stress could cause redox imbalance and upregulate a majority of the pathways of pro-inflammatory mediators [55, 56]. Mitochondria in many cells produce ROS under normal conditions, and the activation of the NLRP3 inflammasome is usually associated with the process of ROS production, which is an essential process for antibacterial cellular activity [57, 58]. Important results demonstrate the role of ROS in NLRP3 activation and that excess ROS production will lead to disturbances [59]. Although the mechanism by which mitochondrial ROS (mROS) stimulates inflammasome activation remains unclear, it may be related to caspase-1-like mROS-dependent redox effects in NLRP3 [60]. One of the mechanisms leading to sarcopenia is the release of mtDNA into the cytoplasm [61, 62]. MROS may oxidize mtDNA and further damage mitochondria and other organelles, releasing more inflammatory factors and mtDNA in the cytoplasm, causing greater damage [4, 39]. Under the acute exercise, the body produces large amounts of ROS, and Astaxanthin exerts a protective effect through its powerful antioxidant activity, reducing oxidative damage and inflammation in muscle and fat, which may prevent sarcopenia in obesity [63].

### Research status of obesity and mtDNA

Studies have shown that overexpression of DsbA-L significantly reduces the release of mtDNA and inflammatory cytokines induced by obesity, thus preventing chronic low-grade inflammation and reducing the occurrence and development of chronic diseases. It indicates that obesity affects the metabolic activity of the whole body through the chronic inflammatory pathway [62]. A critical study found that abnormal mtDNA haplotypes profoundly affected the production of mitochondrial proteases and mROS, insulin signaling, obesity, and aging parameters, including telomere shortening and mitochondrial dysfunction, in a mouse model. It further verified that the functional and structural integrity of mtDNA is closely related to obesity and sarcopenia, and more closely related to the metabolic stability of the whole internal environment [7, 64]. The progressive popularity of metabolic surgery, Roux-en-Y gastric bypass or biliopancreatic diversion, induces weight loss in obese humans and is simultaneously able to reconstitute mitochondrial function after surgery [65]. A significant part of the overall effect of mtDNA number on type 2 diabetes and sarcopenia is mediated by obesity [66]. Clinical data from 14,176 individuals showed that mtDNA quantity was inversely associated with a high risk of metabolic diseases. Obesity and muscle research are gradually increasing, and more evidence has revealed that obesity sarcopenia is a disease closely related to mtDNA. Whether targeting mtDNA can treat obesity sarcopenia needs further explored and proven. The treatment with 5,7-dimethoxyflavone (DMF) significantly increased the downregulation of PGC-1 in the muscle atrophy group. The mRNA expression of NRF-1 and TFAM can also increase the mtDNA content in muscle in a dose-dependent manner, and DMF treatment inactivated the NF-κB pathway to prevent autocrine and paracrine signaling of TNF-α and IL-6, which slowed down the chronic inflammatory effect [67]. It can be inferred that the decrease in pro-inflammatory cytokines after DMF treatment improves PI3K/Akt pathway and stimulates mitochondrial biogenesis [67, 68].

### The mechanism summary of obesity and mtDNA

In the context of obesity, insulin resistance can lead to dysregulation of the Akt signaling pathway, which in turn can contribute to a chronic pro-inflammatory environment [69, 70]. This can have a number of negative effects on mitochondrial function, including damage to mtDNA through various mechanisms. One such mechanism is the disruption of energy uptake at the cellular level, which can lead to increased oxidative stress and damage to mtDNA [71]. Additionally, inflammatory factors can directly attack mtDNA and exacerbate damage [39]. Furthermore, obesity is also associated with accelerated aging, which can further contribute to mtDNA damage [2, 8]. As mtDNA is particularly susceptible to oxidative damage, increased levels of oxidative stress due to obesity and aging can lead to a higher rate of mtDNA mutations and deletions [72, 73]. Therefore, Insulin resistance signaling pathway and inflammatory signaling pathway are the main pathways, such as AMPK, PGC-1α, NLRP3, STING and TGF-β, which affect mitochondrial homeostasis and glycogen consumption, thus forming inflammation and obese sarcopenia from the perspective of mitochondria [40, 61, 62, 68, 74, 75].

To counteract these damaging effects, the body has several mechanisms for repairing mtDNA. One such mechanism is the base excision repair (BER) pathway, which can repair single nucleotide lesions caused by oxidative damage [76]. Another mechanism is the mitochondrial nucleotide excision repair pathway, which can repair bulky DNA lesions caused by UV radiation or chemical exposure [77]. However, in the context of obesity, these repair mechanisms may be compromised due to the chronic inflammatory state and impaired mitochondrial function [68, 78, 79]. This can lead to a cycle of increasing mtDNA damage and decreased repair capacity, further exacerbating the negative effects of obesity on mitochondrial function and overall health.

### Research status of mtDNA and obese Sarcopenia

According to the results and analysis of various studies, the disorder of mtDNA can simultaneously lead to the reduction of muscle mass, the decline of muscle function and the increase of fat, which indirectly reveals that mtDNA can interact with obese sarcopenia [31, 62, 78]. A study quantified mtDNA deletion and copy number using digital PCR techniques to investigate the relationship between skeletal muscle mtDNA copy number, mtDNA deletion mutation frequency, and physical performance indicators in non-diseased older people and demonstrated that mtDNA deletion frequency predicted the decline in physical performance with age, which also provides a new research hotspot for future studies [3, 79–81]. However, only a few researchers have proved the connection between mtDNA and obese sarcopenia [31]. Therefore, there is a lot of research space to explore mitochondrial mysteries, which could be connect with aging, inflammation and healthy maintenance.

## Mitophagy

### Mitophagy is associated with Sarcopenia

The skeletal muscles do not divide because of their final differentiation pattern. As aging progresses, the number of damaged mitochondria increases in cells and cannot be reduced through mitochondrial division and mitophagy [82, 83]. Mitochondrial dysfunction in obesity is reflected by reduced mitochondrial content and impaired function, and existing studies have found decreased enzyme activity in the mitochondria of obese patients, which affects normal mitochondrial metabolism [24, 84]. Autophagy, as a survival mechanism, provides metabolic substrates, such as amino acids, by degrading a large number of cytoplasmic components to cope with hunger [52].

Such is the case with mitochondrial autophagy. However, abnormal self-degradation can lead to abnormal cell death and pathological phenotypes. Therefore, studies of mitochondrial autophagy and metabolic balance have attracted attention [85]. Mitochondrial autophagy is a special form of autophagy in which damaged mitochondria are selectively cleared by the autophagic lysosome system, decomposed into substrates for reuse in cells for synthesis, and would also contribute to energy supply [86]. The autophagic lysosomal function of aged skeletal muscles is generally considered defective. Evidence showed that the loss of *ATG7*, an essential regulator of autophagy, in skeletal muscle leads to bone atrophy and shortened life span [52, 87]. However, overexpression of *ATG7* for 14 days leads to autophagy recovery and an increase in muscle fiber size in aged skeletal muscles [88]. Mitochondrial phagocytosis is crucial for skeletal muscle mitochondria, which can renew metabolism. Mitochondrial phagocytosis maintains a balance under cellular stimulation or physiological conditions. During starvation, mitochondrial phagocytosis occurs to provide the necessary nutrients. Mice with muscle-specific Atg7 deficiency show sharp atrophy of skeletal muscle, a decline in muscle strength, accumulation of abnormal mitochondria and protein aggregates, and increased oxidative stress and apoptosis.[86].

### Research status of mitophagy and sarcopenia

Some studies have shown that reducing the expression of genes involved in mitochondrial phagocytosis leads to mitochondrial dysfunction. Aging-related autophagic changes may lead to the accumulation of damaged macromolecules and organelles. However, the molecular mechanisms regulating autophagy are only partially understood [89]. Therefore, further research is needed to improve our understanding of the exact role of autophagy in skeletal muscle aging [90, 91]. Some studies have proved that mitochondrial autophagy is closely related to lipid metabolism, and specific regulation of genes regulating mitochondrial autophagy will be able to alleviate chronic inflammation and lipid metabolism imbalance caused by the high-fat diet obesity model [78, 88]. Mice lacking Fundc1, a newly discovered mitophagy receptor, will experience more severe obesity and insulin resistance when fed a high-fat diet (HFD) and lead to mitochondrial phagocytosis defect and mitochondrial QC damage in vitro and white adipose tissue (WAT) [78].

In summary, mitochondrial autophagy plays a crucial role in maintaining healthy skeletal muscle and adipose tissue, and this balance can threaten normal health. However, during obesity, mitochondria in the skeletal muscle would promote the progression of autophagy and mitophagy [38]. From the perspective of mitochondrial autophagy, no research has been conducted on obese myocytopenia, and further exploration is needed to explore these associations.

### Research status and mechanism summary of mitophagy and obesity

Under adverse conditions, mitochondrial outer membrane permeability (MOMP) can induce cell death. MOMP is critical for initiating mitochondrial apoptosis and autophagy. When the integrity of the OMM is destroyed, the space proteins between the membranes, cytochrome c, and mtDNA are released into the cytoplasm, thus activating the caspase pathway and apoptosis. One study showed that targeting the cGAS–cGAMP–STING pathway in adipose tissue may effectively improve chronic inflammation caused by obesity and related metabolic disorders (such as sarcopenia) [46, 90]. Because of the chronic inflammation in the cells of obese individuals, mitochondrial metabolism disorder signal factors were observed, revealing that obesity and other adverse factors can also lead to an autophagy balance disorder [78]. Defective mitogenesis leads to persistent and unresolved activation of the NLRP3 inflammasome, leading to autoinflammatory and metabolic disorders that exacerbate the development of metabolic diseases [89]. It showed that the connection between chronic inflammation and skeletal muscle is not only reflected in the abnormality of mtDNA but can also be mediated by mitochondrial autophagy disorder, which proves the relationship between mitochondria and obese sarcopenia.

### Research status and mechanism summary of mitophagy and obese Sarcopenia

Previous studies have shown that the endoplasmic reticulum (ER) and mitochondria are physically connected, forming junctions called mitochondria-associated membranes (MAMs). Since then, some researchers have successfully isolated the MAM fraction and confirmed that MAMs are composed of membrane fragments of the ER and outer mitochondrial membrane (OMM) [34, 46, 92]. In addition, evidence suggested that MAMs are associated with high levels of inflammation, including T2D, obese sarcopenia, and neurodegenerative diseases. As membrane rupture of MAMs leads to internal inflammation, MAMs are the center of the formation of inflammatory bodies, which further proves that mitochondria can stimulate chronic inflammation, leading to further damage, including mtDNA-dependent activation of cGAS STING signaling and mitochondrial autophagy disorder [33, 81, 93].

### Treatment of obese sarcopenia through mitophagy

Considering the information above, autophagy may be related to muscle function. Recently, a study revealed that DMF could relieve skeletal muscle atrophy, and concomitantly, the transcript patterns of biomarkers related to the autophagy–lysosomal system, including Beclin1, LC3, autophagy-related protein (Atg)4, and Atg7, is significantly reduced in sarcopenic groups [67]. Silk Peptide (SP), a beneficial dietary supplement for the prevention of obesity in association with sarcopenia, can promote mitochondrial biogenesis [94]. Exercise is the best non-pharmacological strategy for reactivating mitosis, reducing the accumulation of ROS, thereby improving mitochondrial health and delaying the loss of muscle mass that accompanies various pathologies, including senile sarcopenia, heart failure, and neurological myopathies [34, 95]. Therefore, the selective removal of defective mitochondria by mitosis is key to protecting muscle function.

## Mitochondrial dynamic

### Mitochondrial dynamic disorder mechanism and sarcopenia

Mitochondrial dynamics include division and fusion, related to gene expression and translation. Under normal circumstances, mitochondria are in the process of constant change to adapt to the changing needs of the external environment. Mitochondrial fusion proteins participate in mitochondrial fusion, including mitochondrial fusion proteins 1 and 2 (Mfn1 and Mfn2), which are anchored on the OMM and play a role in signal transduction. Mitochondrial fusion allows the sharing of mitochondrial material, promotes the expansion of the mitochondrial network, and maximizes resource utilization [96]. In contrast, mitochondrial fission decomposes the reticular mitochondria into the smaller, fragmented, single mitochondrion. Mitochondrial fission protein dynamics-related protein 1 (Drp1) resides in the OMM and acts together with mitochondrial fission factor (Mff) and fission protein 1 (Fis1) after activation to promote mitochondrial segregation [97].

Mitochondrial dynamics can regulate the shape of cell organelles, metabolic plasticity, redox homeostasis, and cell survival. Mitochondrial fusion merges the organelle network, mtDNA, and metabolites, and the signals of different mitochondria are cross-linked. However, the mitochondrial division maintains proper allocation of organ resources; simultaneously, the damaged mitochondrial DNA is gathered and then transferred to the lysosome, providing raw materials for synthesizing new substances [93]. Ovariectomized (OVX) animals gain weight, lose weight, and reduce energy consumption. Although a preclinical model of sarcopenia cannot accurately reproduce age-related sarcopenia due to certain limitations, the research mechanism shows that it is related to the impairment of mitochondrial function and the reduction of muscle production, and clarifies the relationship between the dynamics of mitochondria and skeletal muscle [98, 99]. According to the degree of dynamic induction of mitochondria and the physiological environment of skeletal muscle, it can protect or destroy muscle cells.

### Dynamic disorder associated with metabolism will affect skeletal muscle function

Mitochondrial dynamics are regulated by joint fusion and fission cycles performed by complex molecular mechanisms, in which fusion allows connected mitochondria to mix their contents; redistribute metabolites, proteins, and mtDNA and balance the concentrations of various nuclear-coded proteins in each mitochondrion, which Maintain homeostasis of skeletal muscle and related metabolic activities [100–103]. The expanded mitochondrial network is associated with enhanced metabolic function, and disruption of the mitochondrial network is associated with mitochondrial dysfunction and chronic inflammation-related diseases. Evidence suggested that the morphology and dynamics of mitochondria are affected by chronic inflammatory conditions (such as obesity) differently from chronic inflammation-induced sarcopenia [93, 94].

Aging-associated mitochondrial division without damage seems to be processed into the expression of mitophagy proteins, such as Drp1, which increases the ratio of division to fusion. In cells with broken mitochondrial network segments, mitochondria volume in the SS and IMF was reduced, respiration was impaired, and ROS emission was increased. Maintaining mitochondrial morphology is very important for maintaining muscle function and health because the dysfunction of mitochondrial morphology leads to a large number of mROS, affecting the whole body’s metabolic activities [90]. Age-related mitochondrial dysfunction leads to impairment of bioenergy supply. A substantial amount of evidence shows that mitochondrial mass, TCA enzyme activity, and ATP synthesis ability decrease during aging, which also provides new affirmation for skeletal muscle weakness in the aging population. The occurrence and progression of mitochondrial dysfunction are easily affected by the external environment and are also an intersection of many diseases. Various interrelated factors can affect the function of muscle mitochondria throughout life, including internal muscle aging, living habits, chronic inflammation, vascular dysfunction, and hormone changes, which also impart new insights for disease diagnosis and treatment [104]. This research has implications for regeneration therapies in sarcopenia because mitochondrial fission occurs less frequently in satellite cells in older humans [100]. In conclusion, many gaps remain in research on the abnormal dynamics of mitochondria that can occur in patients with obesity sarcopenia, which could be considered as a predictor of disease onset.

### Research status and mechanism summary of mitochondrial dynamics and obese Sarcopenia

Under normal circumstances, this is necessary to remove dysfunctional mitochondria from the mitochondrial pool, which maintains the normal function of mitochondria in cells and the balance of metabolism [91]. These two dynamic modes jointly serve the regular operation of mitochondria and ensure the timely supply and exchange of energy. Previous studies indicate that in the overweight patients tested, the level of MFN2 mRNA focused on the fusion mechanism remained unchanged, but the level of DRP1 expression was significantly reduced. The fusion mechanism was also affected by being overweight, and the protein levels of DRP1 and FIS1 decreased. Therefore, obesity appears to affect the regulation of mitochondrial dynamics during aging, destroying the usual mechanism of division and fusion, which affects its balance [105]. One study showed that diet-induced obesity could promote adipose tissue fibrosis, inflammation, and muscle lipid infiltration in aged rats due to changes in mitochondrial dynamics, which would cause skeletal muscle atrophy and lead to obese sarcopenia [106]. A recent report investigated the physiological role of the post-transcriptional regulation of MFF in skeletal muscle. The RNA-binding protein PUM2 binds and represses the translation of MFF mRNA, which affects fission. In worms, mice, and humans, the binding protein PUM2 increase with age, whereas MFF decreases with age, suggesting that abnormalities in mitochondrial fission and mitosis occurring during aging may contribute to age-related sarcopenia [90].

### Treatment of obese sarcopenia through mitochondrial dynamic

Resveratrol administration attenuated these morphological changes. Further analysis of fluorescence intensity showed that Resveratrol prevented the abnormal reduction in mitochondrial mass, and the treatment could improve the function and quality of skeletal muscle and slow down muscle atrophy [74, 107]. Recently, more and more studies have shown that drug/nutritional food grade L-citrulline and watermelon extract supplements can increase mitochondrial biogenesis, improve adipose tissue polysaccharide, and alleviate skeletal muscle atrophy [108]. *Lactobacillus casei* Shirota and *Lactobacillus paracasei* PS23 can alleviate age-related mitochondrial diseases and inflammation, balance ROS, enhance protein digestibility, and increase the number of mitochondrial copies, thereby increasing muscle mass and function. This clearly reveals that the dynamic nature of mitochondria can be beneficially induced to produce positive changes in skeletal muscle [109]. A recent study showed that the clinical treatment with branched-chain amino acid-enriched mixtures (BCAAem) better impacts mitochondrial function, biogenesis, fusion, and oxidative stress. In animal models, the application of BCAAs has been proven to increase the number and function of the mitochondria effectively [110].

In summary, mitochondrial dynamics can be beneficially induced through different methods, such as small molecule compounds, polypeptides, and probiotics. It is not difficult to find that many scientists have paid attention to the research on mitochondrial dynamics as a target to treat sarcopenia and other metabolic diseases.

## Conclusion

**Fig. 1:**
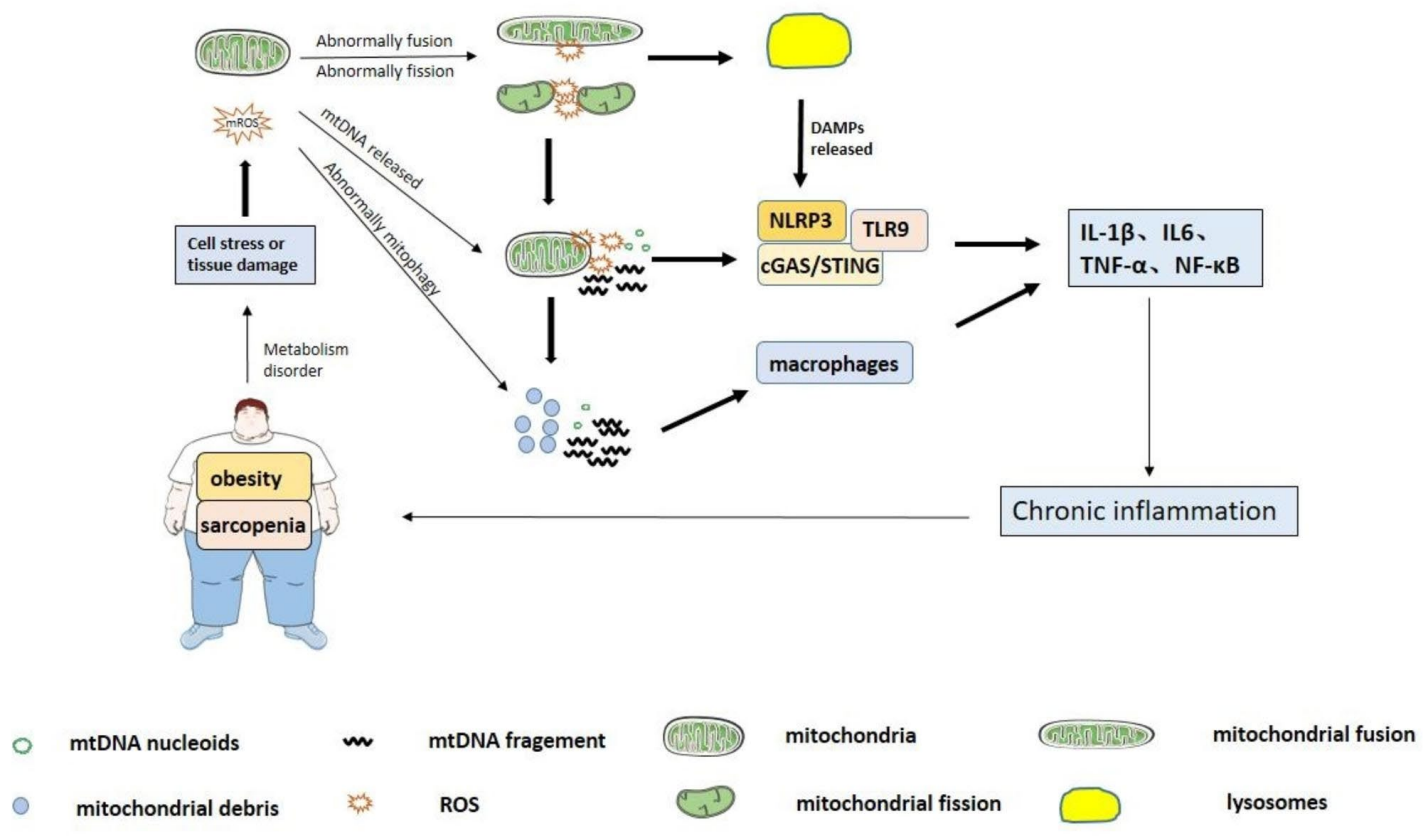
In patients with obese sarcopenia, there is usually tissue damage or cellular stress due to metabolic disorders that eventually lead to mitochondrial disorders: dynamic imbalance, abnormal release of mtDNA and abnormal mitophagy. Among them, the increased dynamic imbalance of mitochondria will intensify the disruption of mitochondrial structure and functions, and the release of mtDNA will lead to abnormal autophagy. During deterioration, dynamic imbalance of mitochondria and abnormal release of mtDNA both stimulate NLRP3, TLR9 and cGAS/STING signaling pathways; the abnormal mitophagy will cause immune stress. Ultimately, mitochondrial disorders will trigger abnormal changes in inflammatory factors, creating long-term chronic inflammation and further exacerbating the development of obese sarcopenia

Skeletal muscle atrophy and obesity are closely related to mitochondrial diseases, and considerable scientific evidence supports this. Mitochondrial diseases can not only serve as the origin of disease development but also as the downstream mechanism of disease occurrence, thus causing a vicious circle, aggravating obese sarcopenia, and causing systemic metabolic disorder, which is more severe than simple sarcopenia (Fig. 1). In current society, many obese patients may not be aware of their combined sarcopenia if their clinician does not perform a muscle assessment. However, these two diseases may occur concomitantly, prolonging the time for disease diagnosis and immediate intervention, requiring attention and awareness of clinicians.

**Fig. 2:**
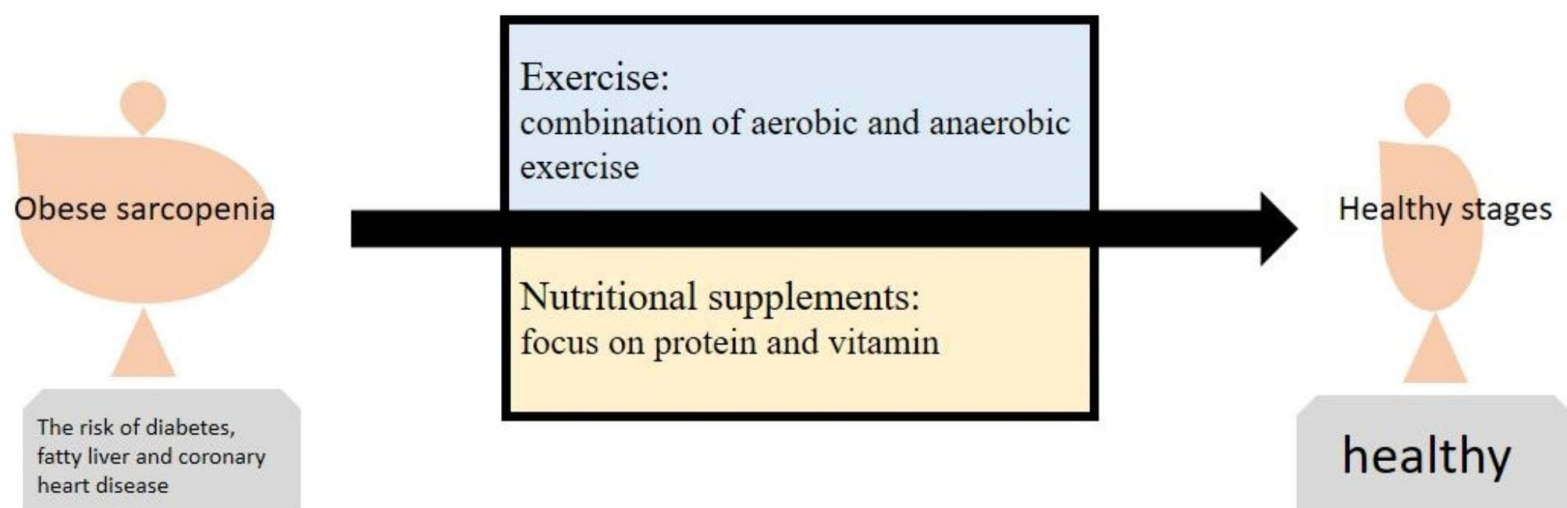
The treatment of obese sarcopenia is recommended to be adjusted accordingly: for nutritional supplementation, it is necessary to focus on the intake of high quality protein; for exercise intervention, it is necessary to combine aerobic and anaerobic exercises to facilitate fat loss and muscle gain of patients

At present, nutritional supplements and exercise interventions are the treatment methods for sarcopenia, which are also effective for obese patients with sarcopenia. For patients with sarcopenia, nutritional supplementation should be combined with a high-protein diet and carbohydrate reduction, while exercise should be combined with aerobic and anaerobic exercise to improve the patient’s internal environment of the body and provide therapeutic benefits (Fig. 2). During the treatment of sarcopenia and obesity, mitochondrial diseases are greatly extensively repaired, indicating that mitochondrial diseases can be used as a test treatment for patient outcomes.

## Data Availability

A statement on how any datasets used can be accessed.
